# Demonstration of the Protein Involvement in Cell Electropermeabilization using Confocal Raman Microspectroscopy

**DOI:** 10.1038/srep40448

**Published:** 2017-01-19

**Authors:** Antoine Azan, Valérie Untereiner, Cyril Gobinet, Ganesh D. Sockalingum, Marie Breton, Olivier Piot, Lluis M. Mir

**Affiliations:** 1 Vectorology and Anticancer Therapies, UMR 8203, CNRS, Gustave Roussy, Univ. Paris-Sud, Université Paris-Saclay, 114 rue Edouard Vaillant, 94805 Villejuif, France; 2 MeDIAN, Biophotonics and Technologies for Health, MEDyC, UMR 7369, CNRS, University of Reims Champagne-Ardenne, 51 rue Cognacq-Jay, 51096 Reims, France; 3 Cellular and Tissular Imaging Platform PICT, Faculty of Pharmacy, University of Reims Champagne-Ardenne, 51 rue Cognacq-Jay, 51096 Reims, France

## Abstract

Confocal Raman microspectroscopy was used to study the interaction between pulsed electric fields and live cells from a molecular point of view in a non-invasive and label-free manner. Raman signatures of live human adipose-derived mesenchymal stem cells exposed or not to pulsed electric fields (8 pulses, 1 000 V/cm, 100 μs, 1 Hz) were acquired at two cellular locations (nucleus and cytoplasm) and two spectral bands (600–1 800 cm^−1^ and 2 800–3 100 cm^−1^). Vibrational modes of proteins (phenylalanine and amide I) and lipids were found to be modified by the electropermeabilization process with a statistically significant difference. The relative magnitude of four phenylalanine peaks decreased in the spectra of the pulsed group. On the contrary, the relative magnitude of the amide I band at 1658 cm^−1^ increased by 40% when comparing pulsed and control group. No difference was found between the control and the pulsed group in the high wavenumber spectral band. Our results reveal the modification of proteins in living cells exposed to pulsed electric fields by means of confocal Raman microspectroscopy.

Electropermeabilization^1^,^2^ (EPN), also known as electroporation, is the destabilisation of the plasma membrane of biological cells caused by intense pulsed electric fields. This destabilisation induces a strong increase in the permeability of the cell membrane, allowing an uptake of external non-permeant molecules by the treated cells. Electrochemotherapy^3^ is one of the major medical applications of cell EPN. It consists in the combination of tumor cells EPN with a chemotherapy (bleomycin or cisplatin) in order to increase the anticancer drug efficiency by a factor ranging from 100 to 1000 depending on the drug. Gene electrotransfer^4^,^5^ is another medical application of the EPN that allows the efficient delivery of plasmid DNA into the cells *in vitro* and *in vivo.*

Because of the complexity of the cell membrane, the underlying mechanisms of EPN are not yet fully understood^6^. It is well established that the electric field induces a transmembrane potential that adds to the resting transmembrane potential of the cell^7^. The membrane permeabilization is triggered as soon as this total transmembrane potential surpasses a certain threshold, which depends on the cell type^8^. Numerical modelling^9^ has shown that the forces induced by the electric field are able to create aqueous pores in the phospholipid bilayer. Although the pores have not been directly visualized in cell membrane^10^,^11^, a computational and experimental study^12^ has demonstrated that siRNA can penetrate into a lipid membrane through the pores created by the intense electric field. Pulsed electric fields are also known to be able to permeabilize the cells for tens of minutes after the delivery of the electric shock^13^ meaning that long term effects are also induced by the EPN process. Atomic Force Microscopy (AFM) performed after the delivery of pulsed electric fields revealed that the membrane elasticity decreased by 40% in live cells^14^. We have shown, by mass spectrometry analysis, that pulsed electric fields initiate chemical reactions at the level of the phospholipids in simple membrane models^15^. Moreover, it has been demonstrated that the presence of modified phospholipids affect the impermeability of the cell membrane^16^,^17^. Therefore, we investigated the potential chemical modifications of cells exposed to pulsed electric fields by confocal Raman microspectroscopy of live cells.

Raman spectroscopy^18^,^19^ is a non-invasive and label-free optical technique that provides detailed information on the molecular composition of the sample. Raman spectroscopy is based on the Raman effect^20^ which consists in an inelastic scattering of light. When a laser beam interacts with a sample, the spectral composition of the scattered light is related to the molecular composition of the sample. This technique has been widely used on biological samples to characterize cells^21^, stem cells differentiation^22^,^23^, drug delivery systems^24^ or to discriminate normal tissues from cancer tissues^25^.

In this study, we have used confocal Raman microspectroscopy to investigate the effect of pulsed electric fields on living cells by comparing the Raman signatures of control and pulsed human adipose-derived Mesenchymal Stem Cells (haMSC). These multipotent adult stem cells are widely used as *in vitro* models^26^,^27^. Their large size and fibroblast-like aspect allows an easy access to the cytoplasmic area far away from the nucleus. Moreover, our laboratory has a strong background on the EPN of haMSC^28^,^29^. The acquisition of the Raman signature of living haMSC was performed for two different spectral ranges and in two different Regions Of Interest (ROI), namely the nucleus and a “cytosolic” nucleus-free area facing the cathode of the generator. The choice of the second ROI was based on fluorescence microscopy *in vitro* studies^8^,^30^ that demonstrated that the strongest effect of pulsed electric fields on the plasma membrane occurred close to the cathode.

## Results

### Fluorescence microscopy of electropermeabilized cells

**Figure 1:**
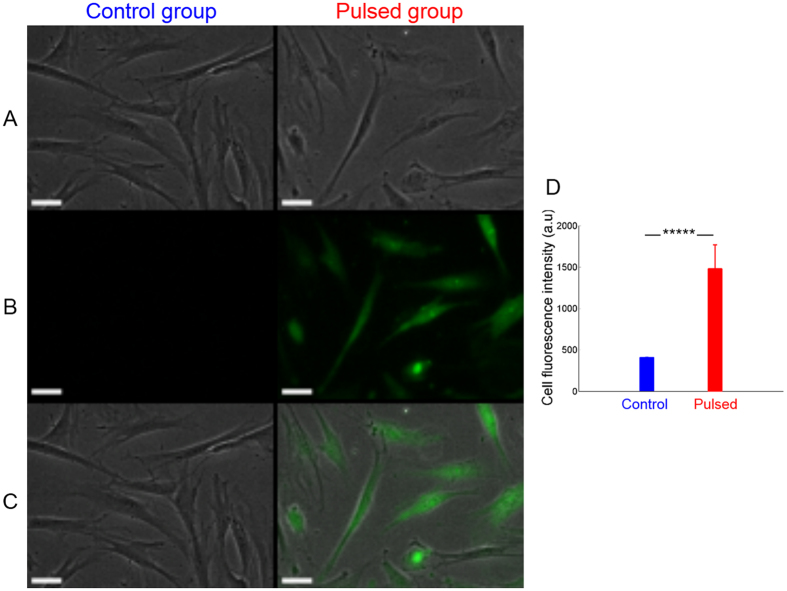
Bright-field and fluorescence microscopy images of control and pulsed haMSC cells. (**A**) – Bright-field images, (**B**) – Yo-Pro-1 fluorescence images, (**C**) – Combined images, (**D**) –Yo-Pro-1 fluorescence intensity into cells. Scale bar: 50 μm. The pulse conditions are 8 pulses, 100 μs, 1 000 V/cm and 1 Hz. The exposure time for fluorescence images was fixed to 200 ms. Images were taken 10 minutes after the delivery of pulsed electric fields. Student’s *t*-test: ****p-value ≤ 0.01%.

Cells were exposed or not to electric pulses (8 pulses, 100 μs, 1 000 V/cm and 1 Hz) in the presence of Yo-Pro-1 fluorescent dye. Figure 1 shows that the fluorescence intensity of the Yo-Pro-1 into inside the cells increased by a factor around 3 between the control group and the pulsed group. This result confirms that haMSC cells have been permeabilized under the condition used for the Raman experiments.

### Micro-Raman analysis of single live cells

**Figure 2:**
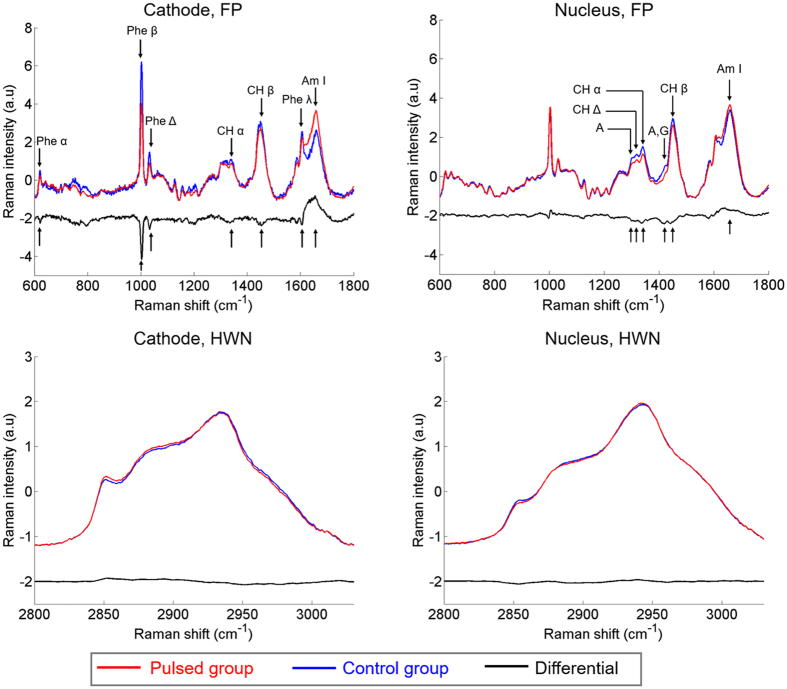
Mean normalized Raman signatures of pulsed and control haMSC cells. The differential spectrum (pulsed group minus control group) is also displayed. For more clarity, differential spectra are displayed with vertical offset. The black arrows indicate the discriminant peaks in the “Cathode, FP” and “Nucleus, FP” conditions. The pulse conditions are 8 pulses, 100 μs, 1 000 V/cm and 1 Hz.

Spectra were acquired at about 12 adjacent positions either in the nucleus area, termed “Nucleus” ROI, or in the “cathodic” part of the cytoplasm, termed “Cathode” ROI (Supplemental Figure S1) for two classical spectral ranges, the FingerPrint (FP) band and the High Wave Number (HWN) band. Figure 2 shows the two mean spectra and the difference spectrum between the pulsed and the control groups for four conditions (“Cathode, FP”, “Nucleus, FP”, “Cathode, HWN” and “Nucleus, HWN”).

As expected^31^, the mean spectrum acquired in the nucleus ROI presents a strong contribution of the DNA/RNA vibrational modes. Among them, the peaks around 783–790 cm^−1^ attributed to the O-P-O backbone stretching of DNA/RNA were observed. The contribution of adenine bands (A) was noteworthy at 1303 and 1425 cm^−1^. The peak at 1425 cm^−1^ was also attributed to the nucleic acids guanine (G) contribution. Protein bands were also present with C-C and C-N stretching modes at 1128 cm^−1^, the phenylalanine (Phe) ring breathing mode at 1003 cm^−1^, and the amide I band (Am I) at 1658 cm^−1^. The “Cathode spectra” were mainly composed of proteins and lipids signatures. In the amide I band, the maximum intensity at 1658 cm^−1^ suggests a predominant α-helix conformation of the proteins^32^. Relatively, the contribution of the phenylalanine peak at 1003 cm^−1^ was higher in the Cathode ROI than in the Nucleus ROI. A tentative band assignment for the haMSC cells spectra based on litterature^31^,^33^,^34^,^35^ is summarized in the Supplementary Information (SI).

Figure 2 shows that the strongest differences between the mean normalized spectrum of the two groups (i.e., control and pulsed) were observed for the “Cathode, FP” condition. Based on the differential spectrum of the “Cathode, FP” condition, seven discriminant bands were identified: 621, 1003, 1033, 1342, 1448, 1607 and 1658 cm^−1^ respectively labelled “Phe α”, “Phe β”, “Phe Δ”, “CH α”, “CH β”, “Phe λ” and “Am I”. As reported in the SI, four discriminant peaks belong to the phenylalanine vibrational modes: 621, 1003, 1033, and 1607 cm^−1^. The 1342 and 1448 cm^−1^ peaks are related to the CH deformation of proteins and lipids, while the 1658 cm^−1^ is assigned to the amide I vibrational mode (C=O stretch). Consistently, the relative magnitude of the four peaks attributed to phenylalanine are decreased when comparing the pulsed group to the control group. No wavenumber shift of the discriminant peaks of phenylalanine was noticed. Thus, we can conclude that the relative concentration of the phenylalanine molecules in the cell decreased or/and that the environment of the phenylalanine molecules was modified after the application of pulsed electric fields. The axial resolution of confocal Raman microspectroscopy did not allow to discriminate the signal contribution from the plasma membrane or from other cellular organelles. But because of its hydrophobic properties, phenylalanine is known to be present in the plasma membrane and to be part of many transmembrane domains^36^. Based on our experience concerning the chemical modifications of phospholipids in the plasma membrane after the delivery of pulsed electric fields, we cannot exclude that chemical modifications of the phenylalanine may be triggered by the EPN process. Our results in the “Cathode, FP” condition show that the relative magnitude of the amide I peak at 1658 cm^−1^ is increased of 40% in the pulsed group with respect to the control group. The amide I band is involved in the peptide bond between consecutive amino acids in the primary structure of proteins. Maiti *et al*. demonstrated that the 1658 cm^−1^ peak is related to the unfolded state of proteins^37^. Recent numerical models have shown that pulsed electric fields can alter the folding of membrane proteins^38^. The evolution of the amide I band may be directly related to the structural modifications of proteins and especially to their folding state. The magnitude of the two vibrational bands attributed to CH deformation (1342 and 1448 cm^−1^) is decreased in the pulsed group compared to the control group. These two vibrational modes are both related to proteins and lipids. This confirms previous results indicating an alteration of lipids in membrane models^15^. Small differences were also noticed around 800 cm^−1^ but remained difficult to attribute to specific bands due to the complex number of vibrational frequencies overlapping in this region. Moreover, the relative differences in these bands were close to the standard deviation of the data set.

For the “Nucleus, FP” condition, the differences were smaller than for the “Cathode, FP” condition. Six discriminant peaks were identified: 1303, 1319, 1342, 1425, 1448 and 1658 cm^−1^ respectively labelled “A”, “CH Δ”, “CH α”, “A, G”, “CH β” and “Am I”. The 1303 and 1425 cm^−1^ peaks belong to the vibrational modes of DNA/RNA. The relative magnitudes of these two vibrational modes attributed to DNA/RNA are decreased in the pulsed cells spectra with respect to the control cells spectra. It is well established that the EPN process induces the generation of Reactive-Oxygen Species (ROS)^39^,^40^. ROS are known to induce strong DNA damages^41^. Moreover, Poplineau *et al*. demonstrated that confocal Raman microspectroscopy is able to detect modifications of DNA in living cells^42^. The decrease of the DNA vibrational peaks observed in the pulsed is consistent with the present literature. The 1319, 1342 and 1448 cm^−1^ peaks are related to the CH deformation of proteins and lipids. Consistently, the magnitude of the three peaks related to CH vibrational modes are decreased when comparing the pulsed group to the control group. The amide I band relative magnitude is increased between the pulsed and control groups. As for the “Cathode, FP” condition, this may be associated to the structural modification of proteins induced by pulsed electric fields. Among these six discriminant peaks of the “Nucleus, FP” condition, three of them (1342, 1448 and 1658 cm^−1^) shared the same evolution in the “Cathode, FP” condition and “Nucleus, FP” condition when comparing the control group and the pulsed group. The modification of the magnitude of CH and amide I bands confirms the strong impact of EPN on the structure of proteins in different cellular organelles.

Contrary to the data in the FP region, the results of HWN region results did not present any obvious difference between the two groups and no clear pattern was observed. Hence, the vibrational modes related to the HWN did not seem to be modified between the control and pulsed groups. The HWN region is mainly composed of CH_2_ and CH_3_ stretching vibrational modes of lipids^43^. The absence of modification in this region would indicate that the lipids were not affected by the pulsed electric fields. Unfortunately, the in-depth resolution of confocal Raman microspectroscopy does not allow to discriminate the signal contribution of the lipids of the plasma membrane from the contribution of the lipids of any cell organelle. Therefore, we cannot exclude that the lipids of the plasma membrane were affected by the pulsed electric fields. Performing the same experiments with a membrane model, Giant Unilamellar Vesicles (GUV), which is a lipid bilayer with a size similar to that of cells^44^, would allow to focus only on the interaction between pulsed electric fields and a lipid bilayer. Acquiring the Raman signatures of GUVs has already been done with a similar confocal Raman microspectroscope^45^. The acquisition of the Raman spectra in the HWN region was also used to monitor the transmembrane potential of cells^46^,^47^. These previous studies have demonstrated that the intensity ratio of the 2930 cm^−1^ peak on the 2850 cm^−1^ peak is related to the transmembrane potential. By calculating this ratio for the “Cathode, HWN” data set, no statistically significant difference appeared between pulsed and control group (data not shown).

Based on this first results, we have investigated the variability and statistical difference of the discriminant peaks identified in the “Cathode, FP” and the “Nucleus, FP” conditions.

**Figure 3:**
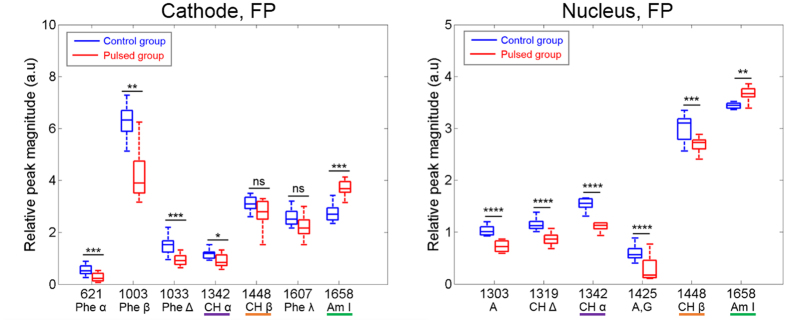
Relative peak magnitude of the discriminant peaks identified of “Cathode, FP” and “Nucleus, FP” conditions. The common bands in the two conditions are underlined with specific color. The p-values between the two groups are displayed. The pulse conditions are 8 pulses, 100 μs, 1 000 V/cm and 1 Hz. Student’s *t*-test: ns (non-statically significant): p-value > 5%, p-value ≤ 5%, **p-value ≤ 1%, ***p-value ≤ 0.1%, ****p-value ≤ 0.01%.

As shown in the “Cathode, FP” panel of Fig. 3 corresponding to the Cathode spectra, the relative magnitude of the four peaks attributed to the phenylalanine amino-acid (i.e., 621, 1003, 1033 and 1607 cm^−1^) is decreased by 30% in the pulsed group versus the control group. Among these four peaks, three of them (621, 1003 and 1033 cm^−1^) showed statistically significant differences between the two groups, supporting the hypothesis of an important effect of pulsed electric fields on the phenylalanine. The 1607 cm^−1^ peak does not show any statistical difference between the two groups. This might be explained by the fact that the 1607 cm^−1^ peak is also attributed to the tryptophan amino-acid which might not be affected by the pulsed electric fields as suggested by the other vibrational modes specific to the tryptophan (762 cm^−1^). The relative magnitude of the amide I band at 1658 cm^−1^ is increased with a statistically significant difference in the pulsed group with respect to the control group. For the CH vibrational modes at 1342 and 1448 cm^−1^, a decrease of 15% is observed when comparing the pulsed group to the control group. This difference is statistically significant for the 1342 cm^−1^ peak but not for the 1448 cm^−1^ peak because of the larger standard deviation due to the predominance of the CH_2_ bending mode of lipids.

For the “Nucleus, FP” condition (Fig. 3), the differences between the two groups were smaller but more statistically significant, than for the “Cathode, FP” condition. All the bands related to CH and CH_2_ bonds (1319, 1342 and 1448 cm^−1^) are decreased by about 20% in the pulsed group with respect to the control group, with a p-value lower than 0.01%. The peaks attributed to DNA/RNA, labeled “A”, and “A, G”, both decreased in the pulsed group compared to the control group. Among the six discriminant peaks identified, the evolution of three of these peaks, 1342, 1448 and 1658 cm^−1^, are similar to that of the “Cathode, FP” analysis but with different p-values. These three peaks are all related to proteins, thus demonstrating that pulsed electric fields strongly affect the proteins present in different cellular compartments.

### Multivariable analysis

In order to confirm these quantitative results, an unsupervised multivariable analysis, Principal Component Analysis (PCA)^48^, was performed for the four different conditions: “Cathode, FP”, “Nucleus, FP”, “Cathode, HWN” and “Nucleus, HWN” (Table 1).

**Figure 4:**
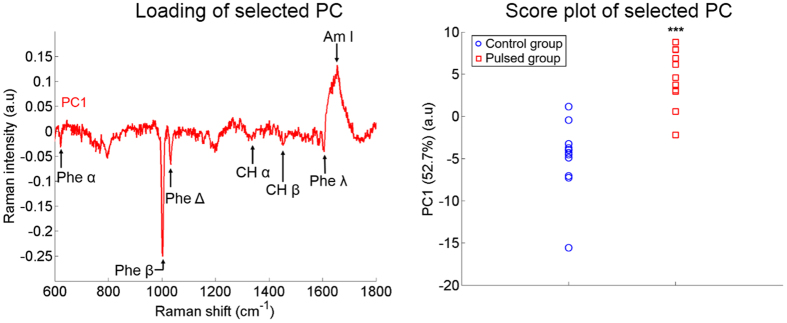
PCA results for the “Cathode, FP” condition. The percentage of variance supported by the selected PC is indicated in brackets. The discriminant peaks identified in Fig. 2 are reported with the same labels on the loading of the selected PC (left). In the score plot, the p-value between the two groups is lower than 0.1% (right). The pulse conditions are 8 pulses, 100 μs, 1 000 V/cm and 1 Hz.

Based on the two selective criteria described in the Materials and Methods section (p-value < 5% and at least 5% of the total variance supported by the selected Principal Components (PCs)), only one PC have been selected for the “Cathode, FP” condition and for the “Nucleus, FP” condition. In the HWN region, none of the PCs fulfills the two criteria. This confirms our previous result demonstrating that the HWN region is not a suitable spectral band to detect any difference in the Raman signature between control cells and pulsed cells. In the case of the “Cathode, FP” condition, the PC1 was selected and accounts for 52.7% of the total variance. PC2 was selected in the “Nucleus, FP” analysis and accounts for 15.3% of the total variance (Fig. 4). This underlines that, for the FP region, the differences between the two groups are stronger in the Cathode ROI than in the Nucleus ROI.

Considering the “Cathode, FP” condition presented in Fig. 4, the selected PC (PC1) obviously shares similar patterns to those of the differential spectrum displayed in Fig. 3. The discriminant peaks identified in the differential spectrum of Fig. 2 are reported with the same labels in Fig. 4. The phenylalanine peaks at 621, 1003 1033 cm^−1^ and around 1200 cm^−1^ contribute to this PC1. The phenylalanine peak at 1003 cm^−1^ is again predominant. Others peaks such as 1658, 1607 1448, 1200 and 800 cm^−1^ are both part of PC1 and of the differential spectrum and share similar relative magnitudes. For this “Cathode, FP” analysis, the correlation coefficient between PC1 and the differential spectrum is equal to 98.6%. When considering the PC1 score (Fig. 4), the difference between the two groups is statistically significant with a p-value lower than 0.1% which means that PC1 seems to be an accurate biomarker of the differences in the Raman signature between pulsed and control haMSC cells.

**Figure 5:**
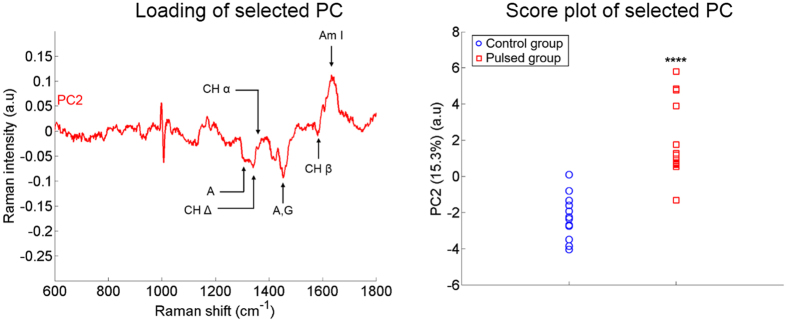
PCA results for the “Nucleus, FP” condition. The percentage of variance supported by the selected PC is indicated in brackets. The discriminant peaks identified in Fig. 2 are reported with the same labels on the loading of the selected PC (left). In the score plot, the p-value between the two groups is lower than 0.01% (right). The pulse conditions are 8 pulses, 100 μs, 1 000 V/cm and 1 Hz.

For the “Nucleus, FP” analysis (Fig. 5), the selected PC (PC2) displays also a pattern similar to the differential spectrum of Fig. 3. The selected PC shows the typical pattern of the shift of the 1003 cm^−1^ peak. The bands at 1303, 1319, 1342, 1425, 1448 and 1658 cm^−1^ are part of both PC2 and the differential spectrum. The relative magnitude of these common bands differs between the two spectra. In PC2, the multiple peaks between 1150 and 1200 cm^−1^ remains difficult to attribute and do not fit with the band assignments reported in the literature. This might be due to the high number of vibrational modes overlapping in this region. The difference between the scores of PC2 of the two groups are statistically significant with a p-value lower than 0.01% (Fig. 5). This confirms our previous analysis showing that the differences in the “Nucleus, FP” condition between the two groups are smaller than in the “Cathode, FP analysis”, but that these differences are statistically more significant.

## Discussion

For the first time, the Raman signatures of living cells submitted to intense pulsed electric fields have been investigated. Our results show that the Raman signature of cells in the FP band is strongly modified by the EPN process. This modification depends on the ROI investigated. For the Cathode ROI, the vibrational modes of phenylalanine, CH chemical bonds of proteins and lipids, as well as amide I are affected by the pulsed electric fields. Peak magnitude analysis demonstrates that the differences between the two groups (control and pulsed) are statistically significant. In the Nucleus ROI, vibrational modes of DNA/RNA, amide I and CH/CH_2_ bonds of proteins and lipids are impacted by the delivery of pulsed electric fields. Acquisition of Raman signature of control and pulsed cells in the HWN spectral band does not show any significant differences. A multivariable analysis by PCA confirmed these results.

This study provides new information at a molecular level on the effect of pulsed electric fields on living cells. This study has revealed significant modifications of phenylalanine and amide I which are directly involved in the protein composition and structure. After the demonstration of the effect of pulsed electric fields on phospholipids using a simple membrane model^15^, our present results show, in a non-invasive and label-free way, that proteins of live cells are also affected by the pulsed electric fields. The axial resolution of confocal Raman microspectroscopy does not allow to discriminate the contribution to the signal coming from the plasma membrane or from other cellular organelles. Acquiring the Raman signature of GUVs and of various ROI of cells, such as the pseudopodia, will allow to investigate deeper the origin of the signal.

## Methods

### Experimental design

**Figure 6:**
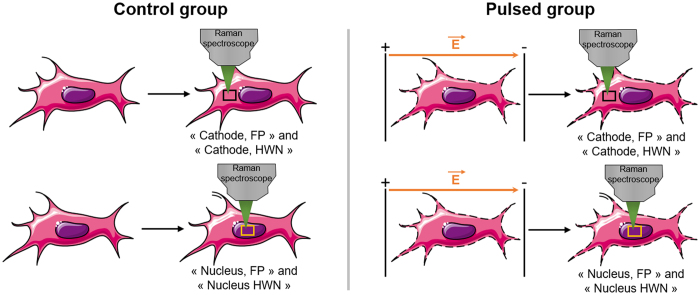
Experimental design for the characterization of control and pulsed haMSC cells by confocal Raman microspectroscopy. The pulse conditions are 8 pulses, 100 μs, 1 000 V/cm and 1 Hz. A 532 nm continuous wave laser of 20 mW is focused on the haMSC cells through a 100 X water immersion objective. In the FP spectral band, the exposure time is fixed to 2 accumulation of 30 seconds. In the HWN spectral band, the exposure time tis fixed to 2 accumulation of 10 seconds. This schematic is adapted from Servier Medical Art.

Cells were exposed or not to pulsed electric fields (8 pulses, 100 μs, 1 000 V/cm and 1 Hz). Control cells underwent sham exposures. The Raman signatures of cells were acquired just after the exposition to pulsed electric fields. Each recorded spectrum was labelled as “control” or “pulsed” based on the delivery or not of pulsed electric fields. In a first experiment, the Raman signature of cells was acquired at a specific ROI and spectral band. The first ROI investigated was an area of the cell between the nucleus and the edge of the cell facing the cathode of the pulse generator (termed here “Cathode”). In another experiment, the Raman signatures at the nuclear compartment of the cells were acquired. Since at this ROI, the nucleus occupies most of the volume, the corresponding Raman spectra were termed “Nucleus”. The differences in the Raman signature between control and pulsed cells were investigated in the FP region (600–1800 cm^−1^) and the HWN region (2600–3100 cm^−1^) for the two specific ROIs (Cathode and Nucleus). In order to limit as much as possible the membrane resealing process following the pulsed electric fields, every experiment was performed at 4 °C, which is known to block the endocytic membrane trafficking^49^,^50^ and causes a “rigidification” of the membrane^51^. It has also been demonstrated that cells can be maintained permeabilized for hours at 4 °C without affecting the cell viability^52^. In total, the Raman signatures of cells were acquired in four different conditions (two ROIs: Cathode and Nucleus and two spectral regions: FP and HWN). Figure 6 summarizes the experimental design.

### Cell culture

haMSC cells were grown in Dulbecco’s Modified Eagle Medium – DMEM - (Life Technologies, Cergy-Pontoise, France) with 10% fetal bovine serum (Life Technologies) and 1% of penicillin-streptomycin (Life Technologies). The cells were maintained in a humidified atmosphere at 37 °C and 5% CO_2_. For the experiment conducted under the confocal Raman microspectroscope, the cells were plated at 5 000 cells/cm^2^ in a 35 mm Petri dish (Thermo Fisher Scientific, Illkirch, France) in which a CaF_2_ window (Crystran, Poole, United Kingdom) was placed at the bottom of the Petri dish before adding the cells. CaF_2_ was chosen because it has been recently proven that a CaF_2_ substrate and a 532 nm laser source is the best combination to acquire Raman signatures of living cells with an optimal quality^53^. After the cells attached to the CaF_2_ window, they were incubated overnight before performing the experiments. No morphological changes were noticed in the cells cultured on this specific substrate. It has been demonstrated previously that CaF_2_ windows are compatible with live cell studies^54^. For this study, haMSC cells were from passage 8 to 11.

### Pulse generator and pulses condition

A commercially available generator (Cliniporator, IGEA, Italy) was used to treat the cells. The pulse conditions were 8 pulses of 100 μs at a magnitude of 1 000 V/cm and a frequency rate of 1 Hz. To deliver the electric pulses on attached cells, a homemade system of electrodes was used. It consisted of two stainless steel plate electrodes and a specific cover that matched with the diameter of the Petri dish. The cover has two slots designed to space the two plate electrodes by 4 mm. When the cover was placed on the Petri dish, the plate electrodes were dropped on the CaF_2_ window through the slots which fixed the distance between the two parallel plates. The plate electrodes were connected to the Cliniporator with alligator clips. Before delivering the pulsed electric fields, the cell culture medium was removed and replaced by a saline solution pre-cooled at 4 °C (B. Braun, Boulogne-Billancourt, France) after washing twice with phosphate-buffered saline. The saline solution, which is a solution of NaCl at 154 mM, was selected as a cell solution because of its low Raman signature and its compatibility with biological cells. No heating effect, pH change or bubble formation have been noticed after the delivery of pulsed electric fields.

### Fluorescence Microscopy

The achievement of cell EPN under strictly the same experimental condition as Raman experiments (5 000 cells/cm^2^ platted overnight on CaF_2_ substrate in 35 mm Petri dish, saline solution maintained at 4 °C, 8 electric pulses of 100 μs duration at a frequency of 1 Hz, magnitude of the electric field of 1 000 V/cm) was checked by fluorescence microscopy^55^. Yo-Pro-1, with λexcitation = 491 nm and λemission = 509 nm, (Life technologies) is a non-permeant intercalating fluorescence dye commonly used to check cell permeabilization^55^,^56^. The level of Yo-Pro-1 fluorescence strongly increases when the dye reaches the nucleus. haMSC cells were platted overnight on a CaF_2_ substrate. Before delivering the pulsed electric field, Yo-Pro-1 was added to the saline solution at a final concentration of 1 μM. Images were acquired with an Observer Z1 inverted microscope (Zeiss, Marly-le-Roi, France) with a LED-based illumination system (Mightex, Toronto, Canada). The LEDs were supplied by a specific power supply system combining three devices (2651 A, 3706AS and 3721) from Keithley (Les Ulis France). Images were acquired with an exposure time fixed to 200 ms for the green channel and 40 ms for the bright-field channel. The lookup table was the same for all the images shown. For the pulsed group, images were acquired 10 minutes after the delivery of pulsed electric fields. In order to quantify the mean level of fluorescence, each cell was isolated on the bright field images. The mean fluorescence per cell was determined with the Zen Blue 2 Zeiss software. The statistical difference between the two groups (control and pulsed) was calculated by Student’s *t*-test.

### Raman Measurements

A confocal Raman microspectrometer LabRam ARAMIS (Horiba Jobin Yvon, Villeneuve d’Ascq, France) coupled with an upright Olympus microscope BX 41 (Olympus, Rungis, France) was used to acquire the Raman spectra of living cells. A 100 X water immersion objective (LUMPLFL 100X/W, Olympus, Rungis, France) was used to focus the laser on the sample and to collect the Raman scattered light. The power of the 532 nm continuous wave laser was around 20 mW at the sample. Similar lasers have been used to acquire the Raman signature of live cells without inducing any phototoxicity^57^,^58^,^59^. The illumination system provided a lateral resolution of 1 μm. The axial resolution was about 7 μm. A 1200 lines/mm diffraction grating was used. It provided a spectral resolution of 1.29 cm^−1^. By rotating the diffraction grating, the FP region (600–1800 cm^−1^) and the HWN region (2600–3100 cm^−1^) were accessible. The sample was placed on an XY piezoelectric stage to investigate various locations.

Prior to any measurement, the confocal Raman microspectroscope was calibrated with a Silicon sample using the 519 cm^−1^ band and the laser power was checked. The acquisition time was fixed to two accumulations of 30 seconds (60 seconds in total) for the FP region and two accumulations of 10 seconds (20 seconds in total) for the HWN region. During each session, the Raman signatures of three to five cells were measured. The measurements were performed consecutively cell by cell with about 10 spots per cell. Due to the laser spot size of 1 μm, the minimum step size was fixed to 2.5 μm in order to avoid any overlapping. Figure S1 displays representative examples of acquisition spots in the living haMSC in the 2 ROIs. No specific cell orientation to the electric field was selected. During the Raman measurements, cells were maintained at 4 °C by the T95 temperature controller (Linkam Scientific Instrument Ltd, Tadworth, UK). Each measurement session lasted one hour maximum. The cells showed no morphological changes during that time period. At the end of the experiment, a Trypan blue test was performed and remained negative, showing that the cells were still alive after one hour under the confocal Raman microspectroscope. No time effect was noticed in the spectral data set, meaning that the first recorded Raman spectrum is similar to the last one, after one hour of Raman signal acquisition.

The confocal Raman microspectroscope was controlled by the LabSpec 5 software (Horiba, Villeneuve d’Ascq, France). Each experiment was independently repeated three times. The Raman signature of the saline solution was acquired in order to be able to remove this interference signal from the measured spectra. In total, 1 318 spectra were collected, attributed to 134 cells. Table 2 details the number of cells per condition. The exposure time in the HWN region is lower than for the FP region. Thus, more cells were investigated in the HWN region than in the FP region within the one-hour duration of the experiment.

### Raman data pre-processing and processing

First of all, each spectrum with a Signal-to-Noise Ratio (SNR) lower than 10 was discarded from the dataset. SNR was calculated on the measured spectra using equation (1):


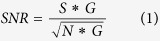


where S is the magnitude of the peak at 1003 cm^−1^, N is the total magnitude of the signal at 1003 cm^−1^ minus the CCD offset and G is the gain of the CCD detector. Less than 5% of the total data set was removed.

Data pre-processing consisted in four steps: Savitsky-Golay smoothing (12 points, 2^nd^ order polynomial)^60^, removing of the saline solution signal, baseline subtraction (8^th^ order polynomial for the FP region, 2^nd^ order polynomial for the HWN region) and normalization by Standard Normal Variance (SNV) method^61^. Each cell was represented by the mean of all its normalized spectra.

For the two groups (control and pulsed), the mean normalized spectrum was calculated. The difference spectrum between the two groups (pulsed group minus control group) was also determined. Based on the difference spectrum, discriminant peaks were identified. Student’s *t*-tests were performed to evaluate the statistical differences between the two groups in the normalized Raman intensity of these discriminant peaks.

Multivariate analyses were performed to quantify the effect of pulsed electric fields on the Raman spectrum of the cells. After mean-centering the data set, PCA was performed on the control and pulsed groups. The PC selection was based on two quantitative criteria that had to be fulfilled. The variance supported by the PC had to be higher than 5% of the total variance of the data set. Otherwise, the risk to take into account a PC attributed to noise variance was non-negligible. Also, the scores of the PC for the two groups had to be statically significant (Student’s *t*-test, p-value < 0.05) in order to consider that this PC was statistically linked to EPN. This data processing was applied for the four conditions (i.e., “Cathode, FP”, “Nucleus, FP”, “Cathode, HWN” and “Nucleus, HWN”). All data processing and data analysis were performed under MATLAB v2009b (MathWorks, Meudon, France).

## Additional Information

**How to cite this article**: Azan, A. *et al*. Demonstration of the Protein Involvement in Cell Electropermeabilization using Confocal Raman Microspectroscopy. *Sci. Rep.* **7**, 40448; doi: 10.1038/srep40448 (2017).

**Publisher's note:** Springer Nature remains neutral with regard to jurisdictional claims in published maps and institutional affiliations.

## Supplementary Material

Supplementary Information

## Figures and Tables

**Table 1:** Comparison of PCA results for the conditions: “Cathode, FP”, “Nucleus, FP”, “Cathode, HWN” and “Nucleus, HWN”.

PC	Cathode, FP		Nucleus, FP		Cathode, HWN		Nucleus, HWN	
	Variance (%)	p-value (%)	Variance (%)	p-value (%)	Variance (%)	p-value (%)	Variance (%)	p-value (%)
1	**52.7**	**<0.1**	61.6	6	84.1	40	82.2	22
2	24.5	49	**15.3**	**<0.01**	7.9	74	n/a	n/a
3	11.7	68	11.2	14	n/a	n/a	n/a	n/a
4	n/a	n/a	n/a	n/a	n/a	n/a	n/a	n/a
5	n/a	n/a	n/a	n/a	n/a	n/a	n/a	n/a

The PCs supporting a total variance percentage lower than 5% are indicated as not applicable (n/a). The p-value is calculated based on the Student’s *t*-test. The PCs fulfilling the two selective criteria (i.e., total variance >5% and p-value ≤ 5%) are indicated in bold.

**Table 2:** Number of cells characterized by confocal Raman microspectroscopy per condition.

		Number of cells characterized	
		Control group	Pulsed group
FP	Cathode	13	12
	Nucleus	12	15
HWN	Cathode	17	22
	Nucleus	23	20
