# Supplementary material for: Demonstration of the Protein Involvement in Cell Electropermeabilization using Confocal Raman Microspectroscopy
Source: Sci Rep. 2017 Jan 19;7:40448. doi: 10.1038/srep40448 (PMC5244372; doi:10.1038/srep40448)

## Demonstration of the Protein Involvement in Cell Electropermeabilization using Confocal Raman Microspectroscopy

Antoine Azan^1^, Valérie Untereiner^2,3^, Cyril Gobinet^2^, Ganesh D. Sockalingum^2^, Marie Breton^1^, Olivier Piot^2,3^, and Lluis M. Mir^1,*^

^1^Vectorology and Anticancer Therapies, UMR 8203, CNRS, Gustave Roussy, Univ. Paris-Sud, Université Paris-Saclay, 114 rue Edouard Vaillant, 94805 Villejuif, France.

^2^MeDIAN, Biophotonics and Technologies for Health, MEDyC, UMR 7369, CNRS, University of Reims Champagne-Ardenne, 51 rue Cognacq-Jay, 51096 Reims, France.

^3^Cellular and Tissular Imaging Platform PICT, Faculty of Pharmacy, University of Reims Champagne-Ardenne, 51 rue Cognacq-Jay, 51096 Reims, France.

## Correspondence and requests for materials should be addressed to Lluis. M. Mir (Luis.Mir@gustaveroussy.fr)

**Supplementary Information**

**Figure S1:** Representative example of acquisition spots in living haMSC in A – Cathode ROI, B – Nucleus ROI. The green dots represent the spots where the Raman signatures were acquired.

**
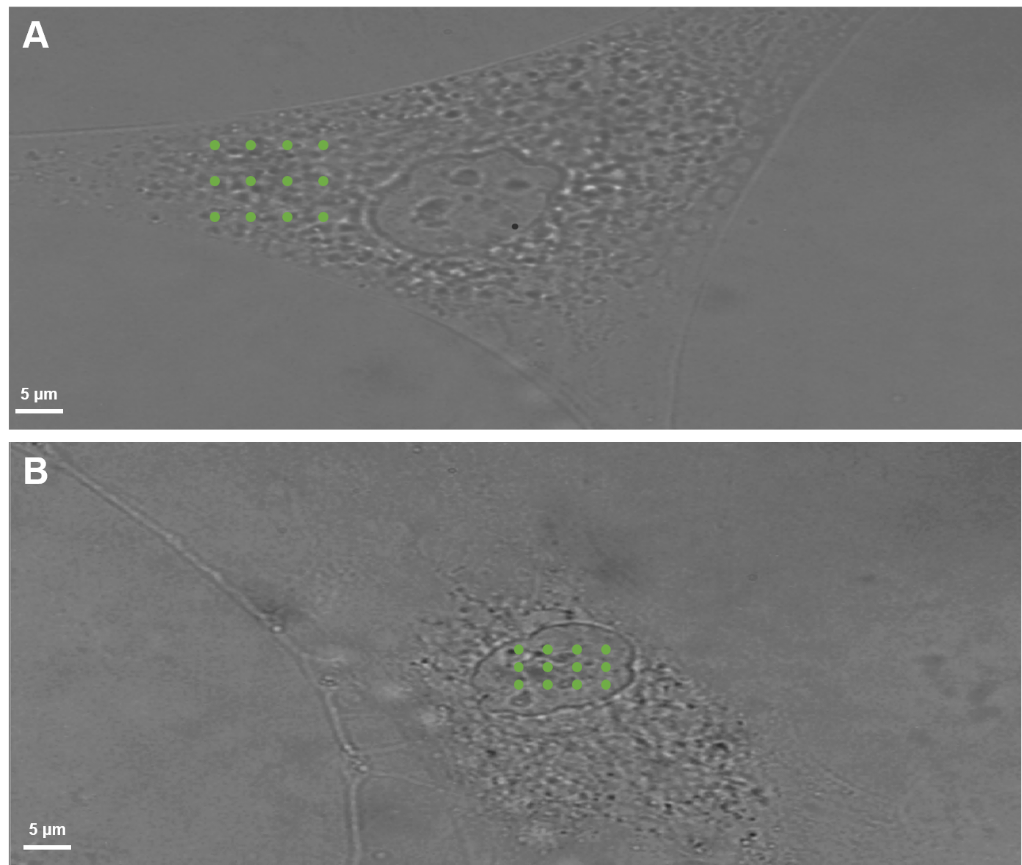
**

**Table S1:** Tentative Raman band assignment of haMSC cells. Abbreviations: A: Adenine, G: Guanine, T: Thymine, C: Cytosine, U: Uracil, Phe: Phenylalanine, Tyr: Tyrosine, Trp: Tryptophan, str: stretching; def: deformation, bend: bending, sym: symmetric, asym


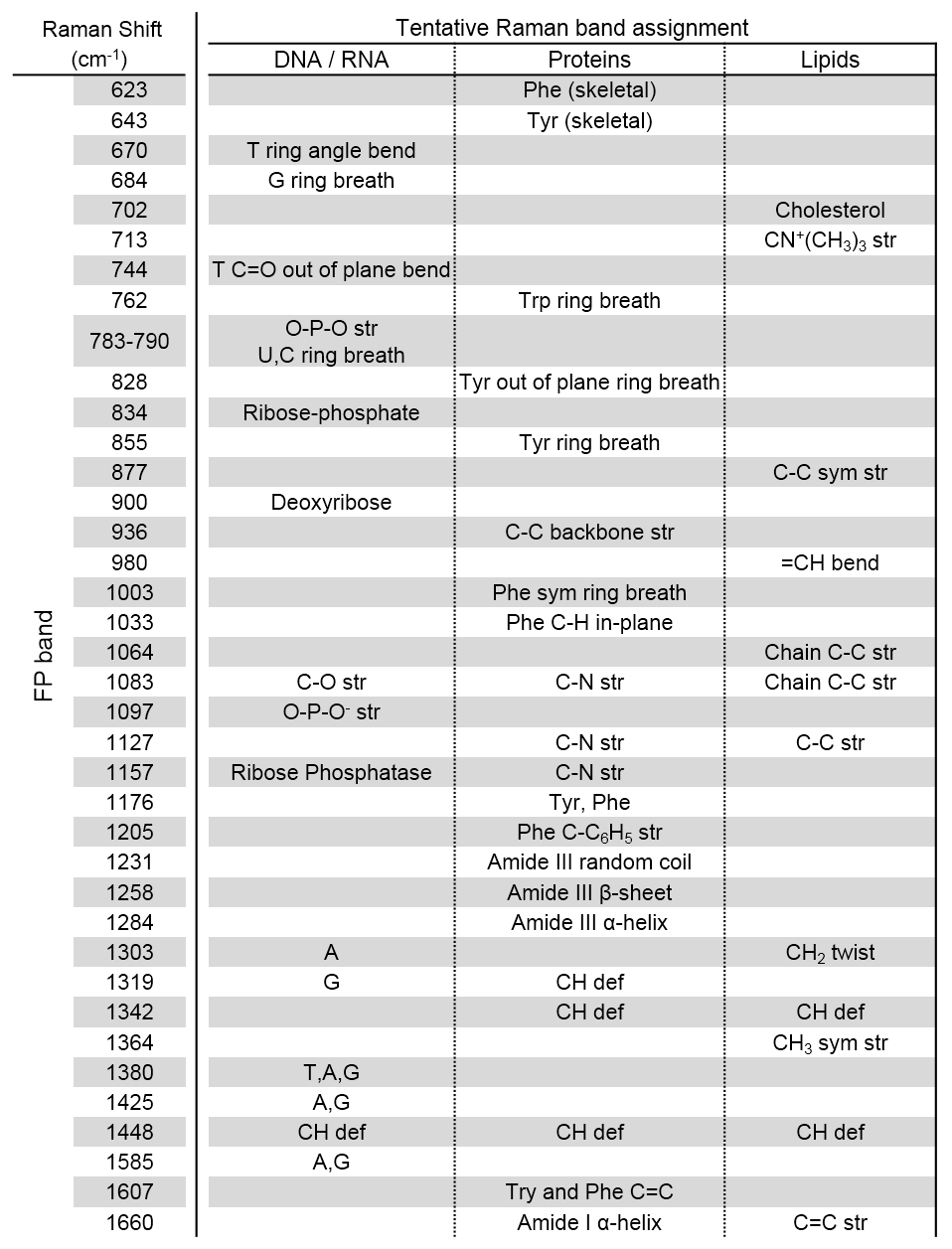

Supplement: Supplementary Information [file srep40448-s1.doc]
